# Association between admission plasma 2-oxoglutarate levels and short-term outcomes in patients with acute heart failure: a prospective cohort study

**DOI:** 10.1186/s10020-019-0078-1

**Published:** 2019-03-28

**Authors:** Zhengliang Peng, Qiong Zhan, Xiangkun Xie, Hanlin Li, Yan Tu, Yujia Bai, Xingfu Huang, Wenyan Lai, Boxin Zhao, Qingchun Zeng, Dingli Xu

**Affiliations:** 1 State Key Laboratory of Organ Failure Research, Department of Cardiology, Nanfang Hospital, Southern Medical University, 1838 Northern Guangzhou Ave, Guangzhou, 510515 Guangdong China; 2 Key Laboratory for Organ Failure Research, Ministry of Education of the People’s Republic of China, Guangzhou, China; 3 Department of Pharmacy,Nanfang Hospital, Rational Medication Evaluation and Drug Delivery Technology Lab, Guangdong Key Laboratory of New Drug Screening, Guangzhou, China

**Keywords:** 2-oxoglutarate, Acute heart failure, Prognosis, Cohort

## Abstract

**Background:**

2-oxoglutarate (2OG), an intermediate metabolite in the tricarboxylic acid cycle, has been found to associate with chronic heart failure (HF), but its effect on short-term adverse outcomes in patients with acute HF (AHF) is uncertain.

**Methods:**

This prospective cohort study included 411 consecutive hospitalized patients with AHF. During hospitalization, fasting plasma samples were collected within the first 24 h of admission. Plasma 2OG levels were measured by hydrophilic interaction liquid chromatography-liquid chromatography tandem mass spectrometry (HILIC-LC/MS/MS). All participants were followed up for six months. Multiple logistic regression was used to determine the odds ratio (OR) and 95% confidence interval (CI) for primary outcomes.

**Results:**

The AHF cohort consisted of HF with preserved ejection fraction (EF) (64.7%), mid-range EF (16.1%), and reduced EF (19.2%), the mean age was 65 (±13) years, and 65.2% were male. Participants were divided into two groups based on median 2OG levels (μg/ml): low group (< 6.0, *n* = 205) and high group (≥6.0, *n* = 206). There was a relatively modest correlation between 2OG and N-terminal pro B-type natriuretic peptide (NT-proBNP) levels (r = 0.25; *p* < 0.001). After adjusting for age, sex, and body mass index, we found that the progression of the NYHA classification was associated with a gradual increase in plasma 2OG levels (p for trend< 0.001). After six months of follow-up, 76 (18.5%) events were identified. A high baseline 2OG level was positively associated with a short-term rehospitalization and all-cause mortality (OR: 2.2, 95% CI 1.3–3.7, *p* = 0.003), even after adjusting for NT-proBNP and estimated glomerular filtration rate (eGFR) (OR: 1.9, 95% CI 1.1–3.4, *p* = 0.032). After a similar multivariable adjustment, the OR was 1.4 (95% CI 1.1–1.7, *p* = 0.018) for a per-SD increase in 2OG level.

**Conclusions:**

High baseline 2OG levels are associated with adverse short-term outcomes in patients with AHF independent of NT-proBNP and eGFR. Hence plasma 2OG measurements may be helpful for risk stratification and treatment monitoring in AHF.

**Trial registration:**

ChiCTR-ROC-17011240. Registered 25 April 2017.

## Background

Acute heart failure (AHF) is a life-threatening syndrome that needs prompt diagnosis and therapy (Mebazaa et al. 2015; Cosentino and Campodonico 2018; Ahmad and Felker 2018). Despite recent advances in the treatment of chronic HF (CHF), the prognosis of AHF remains relatively poor (Mebazaa et al. 2015; Ponikowski et al. 2016). In AHF patients, the composite endpoint of mortality and readmission for HF within six months approached 40% (Felker et al. 2010). Increasing research has shown that HF is associated with metabolic dysfunction (Rame 2012; Cheng et al. 2015; Doehner et al. 2014; Wang and Gupta 2015). Circulating metabolic markers may play an important role in the risk stratification and prognosis assessment of HF, and in the guidance of treatment (Mebazaa et al. 2015; Wang and Gupta 2015; Ibrahim and Januzzi Jr. 2018; Berezin 2018).

2-oxoglutarate (2OG) is an important intermediate metabolite of the tricarboxylic acid (TCA) cycle and glutamine metabolism (Wise et al. 2011; Harris 2015). Intermediate metabolites contribute to generate adenosine triphosphate (ATP) for energy and control cell growth and regeneration by providing precursor and signaling molecules (Doenst et al. 2013). Research suggests that the presence of intermediate metabolites may reveal disease status, predict disease progression, and provide valuable prognostic information (Wang et al. 2017; Liu et al. 2018; Dunn et al. 2007; Maus and Peters 2017; Chen et al. 2014). In previous study, we found that high 2OG level in serum showed a significant association with poor prognosis in CHF patients (Chen et al. 2014). However, the association between plasma 2OG level and the short-term prognosis in patients with AHF is leak of corresponding evidence. Advances in metabolite profiling technology have provided researchers with powerful tools with which to accurately measure 2OG levels (Dunn et al. 2007; Magiera et al. 2013). For example, liquid chromatography with tandem mass spectrometry (LC/MS/MS) showed appropriate specificity, sensitivity, and precision for the measurement of 2OG level and can be applied in clinical practice (Magiera et al. 2013). The advantage of hydrophilic interaction liquid chromatography (HILIC), which enable to enhance the retention of polar analytes (e.g., plasma 2OG) and compatible with mass spectrometry (MS), makes the HILIC-LC/MS/MS a better approach to measure plasma 2OG compare to LC/MS/MS (Cubbon et al. 2010).

Here, we report findings from a cohort study that evaluates the prognostic value of 2OG measurements (by HILIC-LC/MS/MS) in patients with AHF. We aimed to assess the association between plasma 2OG levels and short-term outcomes during a 6-month follow-up in 411 AHF patients.

## Methods

### Study design

This study is from an ongoing prospective cohort study (NFHC-HF1.1), which started to enroll patients with HF from November 2017. It was approved by the Ethics Committee of the Nanfang Hospital of Southern Medical University (approval NO. NFEC-2017-063). The study protocol and informed consent are consistent with scientific and ethical requirements, and written informed consent was obtained from all participants or their legally authorized representatives before the enrollment. The cohort study has been registered in the Chinese Clinical Trial Registry (http://www.chictr.org.cn, ChiCTR-ROC-17011240). This research was conducted in a double-blind fashion. Neither the treating physicians nor the patients knew the results of plasma 2OG measurements.

### Study population

The current study included participants from the NFHC-HF1.1 study during November 2017 to June 2018. The following three criteria had to be met according to the 2016 ESC guidelines (Ponikowski et al. 2016): 1) new onset or worsening symptoms and signs of HF (including dyspnea, edema, and fatigue); 2) NT-proBNP levels ≥300 pg/ml; 3) echocardiographic evidence of systolic or diastolic left ventricular dysfunction. Patients with acute myocardial infarction (AMI), severe renal failure (eGFR< 15 ml/min/1.73 m^2^) or with a life expectancy of < 1 year were excluded.

### Study procedures

During hospitalization, fasting plasma blood samples were collected using EDTA tubes within the first 24 h of admission. Samples were immediately processed by centrifugation at 1500 g for 15 min at 4 °C and frozen at − 80 °C within two hours of venipuncture. At the time of analysis, samples were thawed at room temperature and measured (by HILIC-LC/MS/MS) immediately after preparation.

Clinical data were collected and entered into an electronic data capture (EDC) system (https://study.empoweredc.com/home). Automated electronic data checks were performed to prevent out-of-range or duplicate entries. All data were fully anonymized prior to access by any of the investigators.

We evaluated several covariates associated with adverse prognosis in patients with AHF. Hypertension was defined as a systolic blood pressure of ≥140 mmHg and/or a diastolic pressure of ≥90 mmHg, according to the 2010 Chinese guidelines for the management of hypertension (Liu 2011). Diabetes mellitus (DM) was defined as a fasting blood glucose value of ≥7.0 mmol/L and/or a hemoglobinA1c value of ≥6.5%, per the guidelines for the prevention and control of type 2 diabetes in China (2017 Edition). Atrial fibrillation (AF) was identified by electrocardiogram performed during hospitalization and/or from medical records. Bacterial infections were defined by procalcitonin (PCT) ≥0.20 ng/ml and use of antibiotics (Demissei et al. 2016). Patients with left ventricular ejection fraction (LVEF) in the range of < 40%, 40–49%, ≥50% were defined as HF with reduced EF (HFrEF), mid-range EF (HFmrEF), preserved EF (HFpEF), respectively (Ponikowski et al. 2016). Routine biochemical tests were measured on an automatic analyzer (Roche Diagnostics) at the clinical laboratory of the Nanfang Hospital of Southern Medical University. NT-proBNP was measured using an Elecsys proBNP electrochemiluminescence immunoassay (Elecsys proBNP II, Roche Diagnostics). eGFR was calculated based on the modified glomerular filtration rate estimation equation for Chinese patients (Ma et al. 2006).

Participants were contacted by telephone one month after discharge and subsequently at 3-month intervals.

### Plasma 2OG measurements

Plasma 2OG levels were quantified by HILIC-LC/MS/MS assay. The HILIC-LC-MS/MS system consisted of Agilent 6460 Triple Quadrupole LC-MS/MS systems via an ESI interface as described previously (Nie et al. 2018). Details of the measurement can be found elsewhere (Magiera et al. 2013; Cubbon et al. 2010; Nie et al. 2018). Quality control samples were inserted into every batch of clinical samples with the number above 5% of total samples. Testing personnel of 2OG were blinded to the clinical status of patients, and samples were randomly distributed.

### Short-term outcomes

The primary outcome was a composite of rehospitalization for HF and all-cause mortality. Rehospitalization for HF was defined as an unplanned hospitalization resulting from decompensation of HF (Hicks et al. 2018). If the patient had several hospitalizations, the time of the first hospitalization was counted as the outcome. The outcomes were assessed by independent nurse researchers blinded to the 2OG measurements.

### Statistical analysis

Continuous variables were presented as means and standard deviations (SDs) or median value and interquartile ranges (IQRs), while categorical variables were presented as absolute frequencies and percentages. Kruskal-Wallis rank sum tests and Chi-square tests were used to determine the significant differences between the means and proportions of the groups. In the case of nonlinear distributions, continuous variables were log-transformed for further analyses. Spearman’s correlation was used to examine the associations between the plasma 2OG measurements (log-transformed) and other laboratory measurements. Tests for linear trend were performed by entering the median value of each category of NYHA classification as a continuous variable in the models. Multiple logistic regression was used to determine the odds ratio (OR) and 95% confidence interval (CI) for primary outcome according to the median levels (high group vs. low group), as well as according to the plasma 2OG levels as a continuous variable (per SD increase). Adjustments were made for traditional risk factors [including age, sex, body mass index (BMI), current smoking, DM, AF, hypertension, low-density lipoprotein cholesterol (LDL-C) levels, NT-proBNP levels, eGFR, and medications (including angiotensin-converting enzyme inhibitors/angiotensin receptor blockers (ACEIs/ARBs), beta-blockers)]. Subjects lacking plasma 2OG and NT-proBNP data were excluded, and other missing data were calculated as not available (NA) values without imputation.

Analyses were performed using R version 3.4.3 (R Foundation, Vienna, Austria; http://www.R-project.org) and EmpowerStats (X&Y Solutions, Boston, MA; http://www.empowerstats.com). Statistical significance was defined as *p* < 0.05 (2-sided).

## Results

**Fig. 1:**
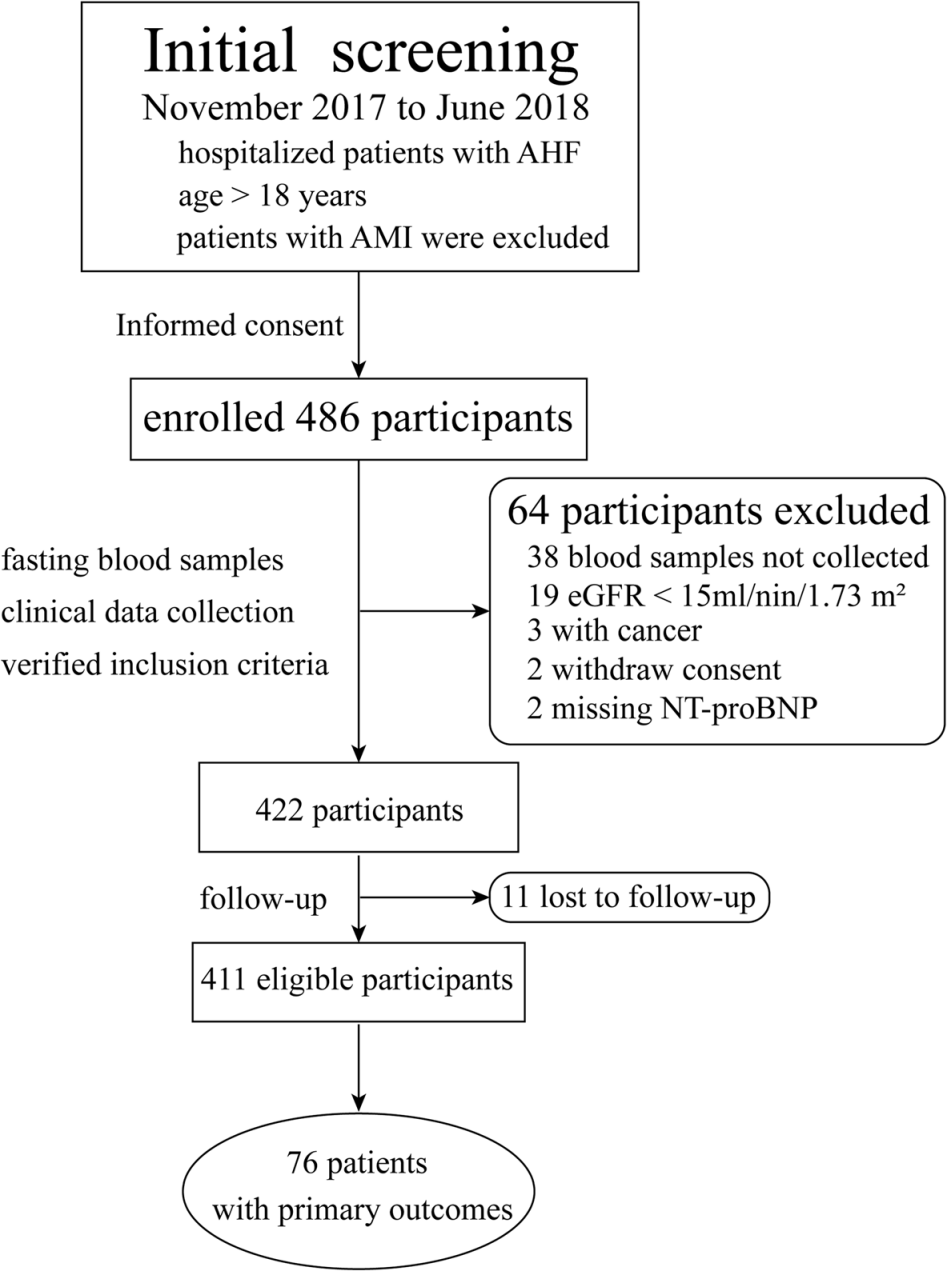
Inclusion flow chart and outcomes for the AHF cohort. AHF: acute heart failure, AMI: acute myocardial infarction, 2OG: 2-oxoglutarate, eGFR: estimated glomerular filtration rate

During the study period, we enrolled 486 participants in the AHF cohort (Fig. 1). After exclusion criteria, a total of 411 eligible participants were included in the final analyses. During the six months of follow-up, 76 (18.5%) events were identified.

### Baseline characteristics

The baseline characteristics of the AHF cohort were shown in Table 1. The mean age was 65 (±13) years, and 65.2% (268/411) were male. The median 2OG level was 6.0 μg/ml (IQR: 4.8–8.1 μg/ml). The types of HF in our cohort were HFpEF (64.7%), HFmEF (16.1%), and HFrEF (19.2%). The most common HF etiology was ischemic heart disease (48.4%). The number of cases with bacterial infection, the most common precipitating factor, was 57 (13.9%). Binary categories, based on the median of the plasma 2OG levels at admission, were 1) low group (*n* = 205 with 2OG < 6.0 μg/ml) and 2) high group (*n* = 206 with 2OG ≥ 6.0 μg/ml).

**Table 1:** Baseline characteristics of the AHF cohort (*n* = 411)

	Low group (*n* = 205)	High group (*n* = 206)	*p*-value
Age, yrs	66.3 (12.0)	63.5 (13.9)	0.027
Sex, male	121 (59.0%)	147 (71.4%)	0.009
Comorbidity			
Current smoking	60 (29.3%)	46 (22.3%)	0.108
Hypertension	129 (62.9%)	99 (48.1%)	0.002
Diabetes mellitus	67 (32.7%)	59 (28.6%)	0.374
COPD	16 (7.8%)	24 (11.7%)	0.188
Atrial fibrillation	55 (26.8%)	70 (34.0%)	0.115
Measurements at admission			
BMI, kg/m^2^	23.9 (3.8)	24.2 (6.8)	0.684
HR, bpm	83.6 (17.5)	87.9 (20.8)	0.026
SBP, mmHg	134.9 (27.1)	125.9 (23.3)	< 0.001
LVEF, %	56.0 (50.0–62.0)	50.5 (39.0–59.0)	< 0.001
eGFR,ml/min/1.73 m^2^	88.2 (64.8–109.8)	86.7 (62.7–106.5)	0.607
Urea, mmol/L	6.3 (5.2–9.1)	6.8 (5.3–9.8)	0.198
UA, μmol/L	405.0 (319.0–489.0)	435.0 (371.2–562.0)	< 0.001
ALB, g/L	^a^ (202) 38.7 (5.0)	(204) 38.8 (5.2)	0.840
TBil, μmol/L	(202) 8.9 (5.7–12.3)	(204) 12.6 (8.1–18.5)	< 0.001
ALT, IU/L	(200) 17.0 (12.0–24.2)	(204) 24.5 (16.0–47.0)	< 0.001
LDL-C, mmol/L	(193) 2.5 (1.9–3.4)	(191) 2.6 (2.0–3.3)	0.372
HDL-C, mmol/L	(193) 1.0 (0.8–1.2)	(191) 1.0 (0.8–1.2)	0.963
CRP, mg/L	(177) 3.9 (1.3–12.0)	(192) 6.1 (1.8–18.4)	0.725
Hb, mg/L	(204) 124.6 (22.6)	(205) 128.7 (23.0)	0.072
NT-proBNP, pg/ml	1257.0 (507.1–3022.0)	2353.5 (841.1–5220.0)	0.008
2OG, μg/ml	4.8 (4.2–5.4)	8.1 (6.7–11.2)	< 0.001
NYHA class			< 0.001
II	121 (59.0%)	79 (38.3%)	
III	56 (27.3%)	80 (38.8%)	
IV	28 (13.7%)	47 (22.8%)	
HF etiology			0.001
Ischemic	108 (52.7%)	91 (44.2%)	
Hypertension	32 (15.6%)	16 (7.8%)	
DCM	16 (7.8%)	34 (16.5%)	
Others	49 (23.9%)	65 (31.6%)	
Type of HF			< 0.001
HFrEF	24 (11.7%)	55 (26.7%)	
HFmEF	26 (12.7%)	40 (19.4%)	
HFpEF	155 (75.6%)	111 (53.9%)	
Bacterial infections	21 (16.0%)	36 (22.1%)	0.192
Medications at discharge			
ACEIs/ARBs	91 (44.4%)	79 (38.3%)	0.214
Beta-blockers	125 (61.0%)	112 (54.4%)	0.175
MRA	80 (39.0%)	112 (54.4%)	0.002
Loop diuretics	89 (43.4%)	126 (61.2%)	< 0.001
Statins	154 (75.1%)	136 (66.0%)	0.043
Outcome			
Primary outcome	26 (12.7%)	50 (21.4%)	0.002
All-cause mortality	8 (3.9%)	17 (8.3%)	0.065

The AHF cohort was divided into two groups according to the median 2OG levels (μg/ml): the low group (< 6.0, *n* = 205) and the high group (≥6.0, *n* = 206). Continuous variables are presented as the mean (SD) or the median with the IQRs (25th, 75th percentiles). Categorical variables are presented as counts and percentiles

Abbreviations: *HF* heart failure, *AHF* acute heart failure, *2OG* 2-Oxoglutarate, *COPD* chronic obstructive pulmonary disease, *BMI* body mass index, *HR* heart rate, *SBP* systolic blood pressure, *LVEF* left ventricular ejection fraction, *eGFR* estimated glomerular filtration rate, *UA* uric acid, *NYHA* New York Heart Association, *ALB* albumin, *TBil* total bilirubin, *ALT* alanine aminotransferase, *LDL-C* low-density lipoprotein cholesterol, *HDL-C* high-density lipoprotein cholesterol, *CRP* C-reaction protein, *Hb* hemoglobin, *NT-proBNP* N-terminal pro B-type natriuretic peptide, *DCM* dilated cardiomyopathy, *HFrEF* HF with reduced ejection fraction, *HFmEF* HF with mid-range ejection fraction, *HFpEF* HF with preserved ejection fraction, *ACEI/ARB* angiotensin-converting enzyme inhibitors/angiotensin receptor blocker, *MRA* mineralocorticoid/aldosterone receptor antagonist. a: the remain valid data regardless of the missing ones

Participants with low 2OG levels tended to be older, with a history of hypertension, and fewer were males (*p* < 0.05). The high group had a lower LVEF than the low group (50.5 (39.0–59.0) vs. 56.0 (50.0–62.0) %, respectively; *p* < 0.001). Correspondingly, HFrEF was more in the high 2OG group (55 (26.7%) vs. 24 (11.7%) in the low group) with a faster baseline heart rate (HR) (87.9 (20.8) vs. 83.6 (17.5) bpm, respectively; *p* = 0.026), lower admission systolic blood pressure (SBP) (125.9 (23.3) vs. 134.9 (27.1) mmHg, respectively; p < 0.001), higher uric acid (UA) levels (435.0 (371.2–562.0) vs. 405.0 (319.0–489.0) μmol/L; p < 0.001), higher alanine aminotransferase (ALT) (24.5 (16.0–47.0) vs. 17.0 (12.0–24.2) IU/L; p < 0.001) and total bilirubin (TBil) levels (12.6 (8.1–18.5) vs. 8.9 (5.7–12.3) μmol/L; p < 0.001). When compared to the low group, the high 2OG group had higher admission NT-proBNP levels (2353.5 (841.1–5220.0) vs. 1257.0 (507.1–3022.0) pg/ml, respectively; *p* = 0.008), and more severe New York Heart Association (NYHA) class (*p* < 0.001). Patients were more likely to have loop diuretics and mineralocorticoid/aldosterone receptor antagonists (MRAs) use at admission in the high 2OG group (*p* < 0.01). In contrast, comorbidity of DM, chronic obstructive pulmonary disease (COPD), and AF, current smoking history, BMI, LDL-C, high-density lipoprotein cholesterol (HDL-C), C-reaction protein (CRP), and eGFR were comparable between the two groups. The usage of ACEI/ARB and beta-blockers at admission in the two groups had no significant difference.

### Relationship between admission plasma 2OG levels and clinical parameters

Among AHF patients, we found a relatively modest but significant correlation between the plasma 2OG and NT-proBNP levels (both measurement results were log-transformed; r = 0.25; *p* < 0.001) and an inverse correlation between the 2OG levels and LVEF (r = − 0.30; p < 0.001). We recorded a significant positive correlation between the plasma 2OG levels and ALT (r = 0.32; p < 0.001), TBil (r = 0.31; p < 0.001), and UA (r = 0.24; p < 0.001).

**Fig. 2:**
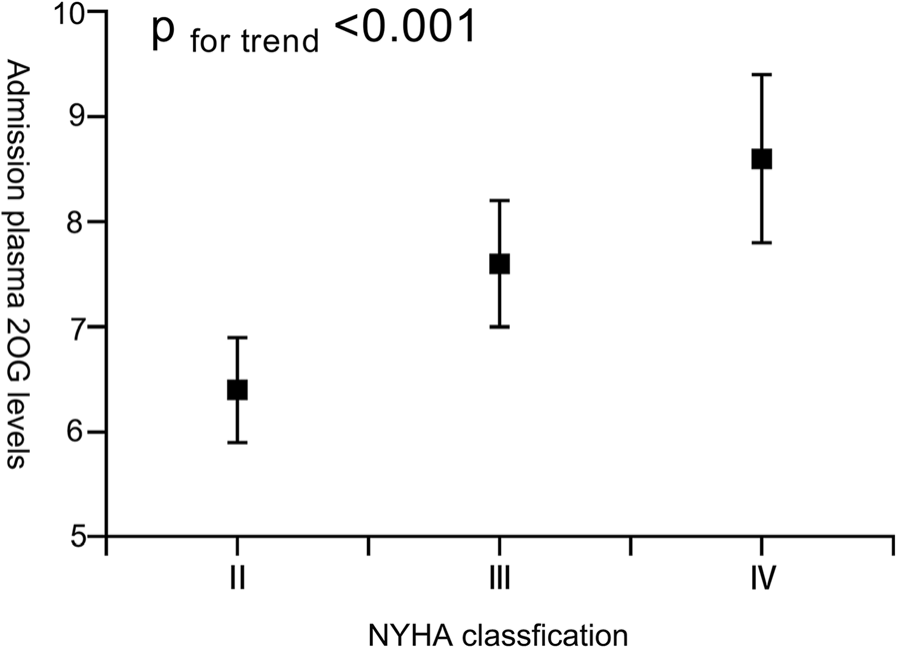
Relationship between the admission plasma 2OG levels and NYHA classification. We calculated the adjusted mean (adjusted for age, sex, and BMI) of 2OG levels for each NYHA classification subgroup. 2OG: 2-oxoglutarate, BMI: body mass index, NYHA: New York Heart Association

We also investigated the relationship between the admission 2OG levels and the NYHA classification. As shown in Fig. 2, with an increase in the NYHA classification, the plasma 2OG levels ascended gradually after adjusting for age, sex, and BMI (p for trend < 0.001).

### Association between admission plasma 2OG levels and short-term outcomes

**Fig. 3:**
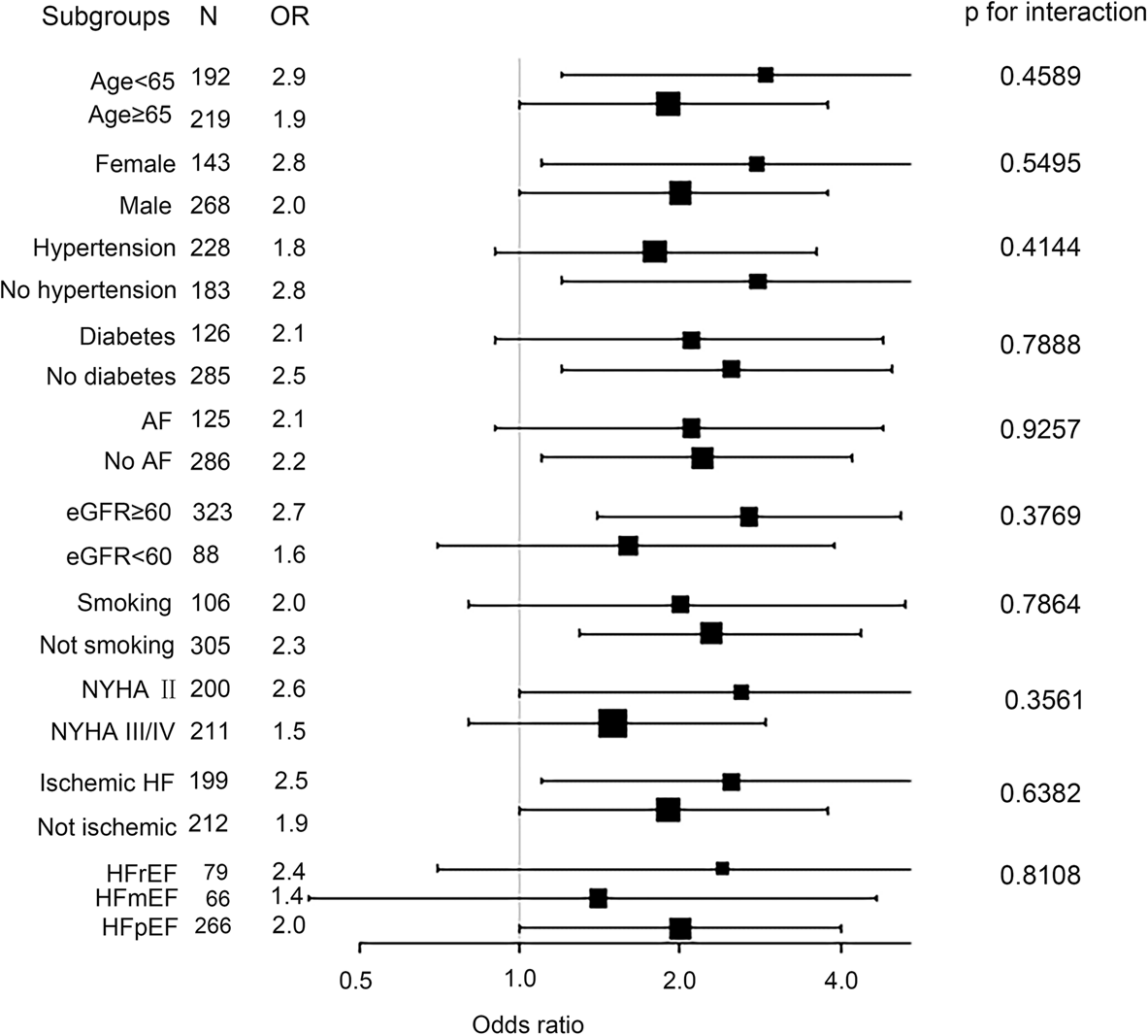
Subgroup analysis of the relationship between the high 2OG group and short-term outcomes. The high 2OG group was associated with adverse short-term outcomes in other clinical subgroups. 2OG: 2-oxoglutarate, NYHA: New York Heart Association, AF: Atrial fibrillation, eGFR: estimated glomerular filtration rate, HFrEF: HF with reduced ejection fraction, HFmEF: HF with mid-range ejection fraction, HFpEF: HF with preserved ejection fraction

During the six months of follow-up, 76 primary endpoints occurred in our AHF cohort. Univariate logistic regression indicated that high admission plasma 2OG levels were associated with increased risk of short-term adverse outcomes, and the OR for the primary outcome was 2.2 (95% CI 1.3–3.7, *p* = 0.003) (Table 2). After adjustment for age, sex, BMI, current smoking, LDL-C, DM, AF and hypertension, the high 2OG group was associated with a twofold increase in risk for primary outcome, OR 2.1 (95% CI 1.2–3.6, *p* = 0.012). We further adjusted for NT-proBNP levels, eGFR and medications. In the final model, patients in the high 2OG group remain had a significantly higher risk of short-term adverse outcomes than those with low admission 2OG levels, OR 1.9 (95% CI 1.1–3.4, *p* = 0.032). As a continuous variable, for a per-SD increase in the admission 2OG level, the OR for primary outcome was 1.4 (95% CI 1.1–1.8, *p* = 0.018) after the similar multivariable adjustment (Table 2). Moreover, risks were similar between the HFrEF and HFpEF subgroups, as well as other clinical subgroups (Fig. 3). There were no significant subgroup interactions.

**Table 2:** Association between the admission 2OG levels and short-term outcomes

	OR (95% CI)	p	Per-SD increase	p
Unadjusted	2.2 (1.3, 3.7)	0.003	1.4 (1.1, 1.8)	0.001
Adjusted model 1	2.1 (1.2, 3.6)	0.012	1.4 (1.1, 1.8)	0.004
Adjusted model 2	1.9 (1.1, 3.4)	0.032	1.4 (1.1, 1.7)	0.018

Model 1: adjusted for age, sex, BMI, current smoking, LDL-C, AF, DM and hypertension; model 2: adjusted for model 1 plus NT-proBNP levels, eGFR, and medications (including ACEIs/ARBs, beta-blockers)

Abbreviations: *2OG* 2-Oxoglutarate, *BMI* body mass index, *LDL-C* low-density lipoprotein cholesterol, *AF* Atrial fibrillation, *DM* Diabetes mellitus, *NT-proBNP* N-terminal pro B-type natriuretic peptide, *eGFR* estimated glomerular filtration rate, *ACEIs/ARBs* angiotensin-converting enzyme inhibitors/angiotensin receptor blockers

## Discussion

We report here a clinical study to investigate the association of admission plasma 2OG levels and short-term outcome in patients with AHF. We found that a high baseline plasma 2OG level was significantly and positively associated with short-term rehospitalization and all-cause mortality, even after adjusting for NT-proBNP and eGFR.

These results help clarify the relationship between plasma 2OG levels and AHF. With the advancements in high-throughput and available metabolomics, mounting evidence suggests that metabolic perturbation is a common trait in HF patients (Cheng et al. 2015; Wang and Gupta 2015; Ussher et al. 2016; Zhang and Abel 2018). Around this scientific problem, several novel terms and concepts generated (Rame 2012; Cheng et al. 2015; Doenst et al. 2013; Zhang and Abel 2018; Neubauer 2007; Chen et al. 2018). The TCA cycle is one of the most fundamental and highly conserved metabolic processes in living organisms including plants, microorganisms, animals and humans (Maus and Peters 2017; Araujo et al. 2014; Huergo and Dixon 2015). As a key intermediate of the TCA cycle, the metabolic implications of 2OG are intriguing.

One decade ago, Dunn and colleagues discovered through data-driven metabolomics approaches that 2OG was a new metabolic biomarker of HF (Dunn et al. 2007). In 2013, Magiera et al. reported that the LC–MS/MS method is an accurate tool for the quantitation of 2OG in human urine samples (Magiera et al. 2013). We subsequently and successfully used the LC-MS/MS method to measure human serum 2OG levels and revealed that a high serum 2OG level was associated with adverse outcomes in CHF patients. The results of our present study are generally consistent with the results we got before, which is a high plasma 2OG level is associated with adverse outcomes in AHF patients. In addition, we used HILIC-LC/MS/MS to measure plasma 2OG levels. With the more accurate measurement method and bigger sample size we enrolled in this study, the consistency between the major findings of our two studies strengthens the conclusion that a high serum/plasma 2OG level is associated with adverse short-term outcomes in both CHF and AHF patients.

We found that the progression of the NYHA classification was associated with a gradual increase in plasma 2OG levels. However, the analysis can only identify relationships between them and cannot determine causation. One controversial issue is whether HF-associated metabolic perturbation is causal or a compensatory adaptation in the process of HF (Zhang and Abel 2018). The current concepts are 1) the heart may have multiple redundant pathways that increase its bioenergetic resiliency and 2) the accumulation of metabolic intermediates may promote structural remodeling (Zhang and Abel 2018). From the perspective of metabolic adaptation, metabolic stress is a characteristic of HF and initiates metabolic remodeling, which may play an important role in the progression of HF beyond ATP and structural remodeling (Doenst et al. 2013; Brown et al. 2017). It will be interesting to explore the relationship between 2OG and HF through well-designed basic experiments. The objective of this study is to obtain a systematic profile of 2OG response to HF by dynamically assaying 2OG and known related metabolites such as citrate, isocitrate, succinate, glutamate, and 2-hydroxyglutarate in HF animal models. Using animal models, we can firstly get samples covering all stages of the process of HF, then, examine whether 2OG changes can modulate the transcriptional and protein expression, and finally investigate the effect of 2OG on HF phenotypes by the intervening 2OG levels.

Although we found that connection of high plasma 2OG levels and poor short-term outcomes in patients with AHF, the mechanisms and significances of plasma 2OG accumulation in AHF remain unclear. Oldham et al. found that in hypoxia-treated cells, most TCA metabolites (including citrate, isocitrate, succinate, fumarate, and malate) were decreased or unchanged, with the exception of 2OG accumulation (Oldham et al. 2015). Interestingly, a previous completed study by Lewis et al. reported that there were no changes in plasma 2OG levels during exercise in an apparent healthy population cohort (Lewis et al. 2010). These data suggest that 2OG accumulation may indicate a disease state and suggest a potential clinical application for plasma 2OG as a biomarker of metabolic stress or metabolic remodeling in disease. The high level of plasma 2OG in AHF patients indicates that metabolic stress or metabolic remodeling may have critical effect on AHF patients which will influence the survival of these patients. These data imply a novel HF-related metabolic mechanism that deserves further exploration.

We demonstrated in the present study that the association between plasma 2OG level and poor prognosis in AHF is independent of eGFR. This finding is consistent with a previous study in which there was no relationship between plasma 2OG levels and renal function markers including urea and creatinine (Dunn et al. 2007). Furthermore, the relationship between plasma 2OG levels and poor short-term outcomes was independent of NT-proBNP levels, despite a modest correlation between 2OG and NT-proBNP. NT-proBNP is an important marker to assess the prognosis of HF patients, and is reflective of the underlying neurohormonal activation and hemodynamic stress, while 2OG may indicate metabolic stress. The relationship between the two biomarkers is worth further study, which is helpful to clarify the pathophysiological mechanism of AHF. Combination of NT-proBNP and eGFR indicating cardiorenal interaction is an important risk factor for adverse outcome in patients with HF. The independent effect of 2OG on the poor prognosis suggests that 2OG-mediated pathways beyond cardiorenal interaction may contribute to adverse short-term outcomes in AHF patients with high plasma 2OG levels.

According to the data analysis, the admission plasma 2OG level was related to abnormal hepatic functions in AHF. These results indicate that liver may plays a major role in 2OG metabolism; Conversely, another explanation is that heart-liver interaction responds to severe HF.

### Study limitations

It is important to note that plasma 2OG levels are responsive to AHF-related *systemic* changes, not the cardiovascular system in isolation. More work is needed to illuminate the mechanisms mediated by 2OG that affect the AHF patients. External validity of these findings is limited because of sample size and population characteristics. Further, this is a prospective study, not a randomized controlled trial, and thus findings are correlational and causation cannot be determined. Last, this study does not provide mechanistic insight into biodynamics of 2OG. Future studies to provide a mechanistic understanding of this metabolite would be beneficial.

## Conclusions

We conclude that for patients with AHF, high baseline plasma 2OG levels are significantly and positively associated with increased short-term rehospitalization and all-cause mortality independent of NT-proBNP and eGFR. A better understanding of 2OG in metabolic remodeling in AHF may shed new light on the mechanism of AHF, and ultimately lead to new therapies for AHF.

## Abbreviations

2OG: 2-Oxoglutarate
ACEI/ARB: Angiotensin-converting enzyme inhibitors/angiotensin receptor blocker
AHF: Acute heart failure
ALB: Albumin
ALT: Alanine aminotransferase
BMI: Body mass index
CI: Confidence interval
COPD: Chronic obstructive pulmonary disease
CRP: C-reaction protein
DCM: Dilated cardiomyopathy
eGFR: estimated glomerular filtration rate
Hb: Hemoglobin
HDL-C: high-density lipoprotein cholesterol
HF: Heart failure
HFmEF: HF with mid-range ejection fraction
HFpEF: HF with preserved ejection fraction
HFrEF: HF with reduced ejection fraction
HR: Heart rate
IQR: Interquartile range
LDL-C: Low-density lipoprotein cholesterol
LVEF: Left ventricular ejection fraction
MRA: Mineralocorticoid/aldosterone receptor antagonist
NT-proBNP: N-terminal pro B-type natriuretic peptide
NYHA: New York Heart Association
OR: Odds ratio
SBP: Systolic blood pressure
SD: Standard deviation
TBil: Total bilirubin
UA: Uric acid
